# Hyperemesis gravidarum and placental dysfunction disorders

**DOI:** 10.1186/s12884-016-1174-7

**Published:** 2016-11-25

**Authors:** Heleen M. Koudijs, Ary I. Savitri, Joyce L. Browne, Dwirani Amelia, Mohammad Baharuddin, Diederick E. Grobbee, Cuno S. P. M. Uiterwaal

**Affiliations:** 1 Julius Center for Health Sciences and Primary Care, Global Health, University Medical Center Utrecht, Huispost Str. 6.131, PO Box 855500, 3508 GA Utrecht, The Netherlands; 2 Budi Kemuliaan Hospital, Jakarta, Indonesia

**Keywords:** *Hyperemesis gravidarum*, *Offspring*, *Placental dysfunction disorders*, *Pregnancy*

## Abstract

**Background:**

Evidence about the consequence of hyperemesis gravidarum (HG) on pregnancy outcomes is still inconclusive. In this study, we evaluated if occurrence of hyperemesis gravidarum is associated with placental dysfunction disorders and neonatal outcomes.

**Methods:**

A prospective cohort study was conducted in a maternal and child health primary care referral center, Budi Kemuliaan Hospital and its branch, in Jakarta, Indonesia. 2252 pregnant women visiting the hospital for regular antenatal care visits from July 2012 until October 2014 were included at their first clinic visit. For women without, with mild and with severe hyperemesis, placental dysfunction disorders (gestational hypertension, preeclampsia (PE), stillbirth, miscarriage), neonatal outcomes (birth weight, small for gestational age (SGA), low birth weight (LBW), Apgar score at 5 min, gestational age at delivery) and placental outcomes (placental weight and placental-weight-to-birth-weight ratio (PW/BW ratio)) were studied.

**Results:**

Compared to newborns of women without hyperemesis, newborns of women with severe hyperemesis had a 172 g lower birth weight in adjusted analysis (95%CI -333.26; -10.18; *p* = 0.04). There were no statistically significant effects on placental dysfunction disorders or other neonatal outcome measures.

**Conclusions:**

The results of our study suggest that hyperemesis gravidarum does not seem to induce placental dysfunction disorders, but does, if severe lead to lower birth weight.

## Background

Nausea and vomiting are common and usually benign symptoms of primarily the first trimester of pregnancy. The onset of nausea correlates with the level of human chorionic gonadotropin (hCG), which typically rises within 4 weeks after the last menstrual period, peaking at approximately 9 weeks of gestation [1]. Sixty percent of nausea cases resolve by the end of the first trimester and 91% by 20 weeks of gestation [1]. Hyperemesis gravidarum is at the severe end of the nausea spectrum and according to The International Statistical Classification of Disease and Related Health Problems (ICD-9), is defined as ‘persistent and excessive vomiting starting before the end of the 22nd week of gestation’. Hyperemesis gravidarum is clinically classified as mild or severe, depending on associated metabolic disturbances such as carbohydrate depletion, dehydration and electrolyte imbalance. Its incidence is estimated at 0.3 to 1.5% of all live births but is unevenly distributed on a global level [2–6]. Asian women, for instance are more likely to suffer from hyperemesis than Caucasian women [3, 5]. Hyperemesis greatly affects maternal well-being and quality of life [7, 8] and is among the most common reasons for pregnancy-associated hospitalization [6, 9, 10].

Relatively little is known about the etiology of hyperemesis [6]. Causal roles of sex hormones, thyroid hormones, *H. pylori* infections and paternal genes have been suggested, although consensus has not been reached [6, 11]. Levels of hCG are positively associated with occurrence and severity of hyperemesis complaints, as seen in multiple- or molar pregnancies [12, 13]. While benign nausea and vomiting in early pregnancy are closely related to temporarily increased hCG levels, it has been argued that in women with hyperemesis, the persistently high hCG level dysregulates normal stimulation of trophoblast migration, which consequently alters placentation [14–16]. Ultimately abnormal placentation could lead to placental dysfunction that clinically manifests as gestational hypertension, preeclampsia, as well as miscarriage, stillbirth and intra-uterine growth restriction (IUGR) [15, 17–19]. In particular, elevated hCG plasma levels in the second trimester are associated with development of these conditions [13, 20]. Thus, hyperemesis gravidarum, occurring in first and early second trimester, could be an early pregnancy indicator of a process that results in symptomatic placental dysfunction later.

There is limited evidence about the consequences of hyperemesis on maternal and offspring’s health. Two large cohort studies in Scandinavian countries showed that hyperemesis was associated with higher risk of preeclampsia, lower birth weight and shorter gestational duration [15, 21]. This was supported by several studies suggesting higher risks of low birth weight (LBW), small for gestational age (SGA), and preterm birth if mothers experienced hyperemesis [22–24]. However, another large study [25] and several smaller studies, [26, 27] did not show such associations.

Women who experience severe hyperemesis have a significantly reduced maternal caloric ‘intake’ and lose additional nutrients and electrolytes [28]. This state resembles fasting and often involves ketonuria, which is frequently tested by clinicians in women suspected of having hyperemesis [29, 30]. Previous studies have shown that placental efficiency changes in women exposed to famine. Increased placental weight in women who were pregnant during the Dutch Hungerwinter suggests that compensatory growth of the placenta can occur in situations where nutritional resources are lacking [31, 32]. The same compensation might occur in women who experience severe hyperemesis gravidarum, however evidence is lacking.

Both hyperemesis and placental dysfunction constitute substantial maternal and neonatal health threats, particularly in the low and middle income countries where health care resources are limited [33]. Therefore, further exploration of a relation between hyperemesis and such disorders is warranted.

## Methods

The present study aimed to investigate the relation between hyperemesis and placental dysfunction disorders (gestational hypertension, preeclampsia, miscarriage, and stillbirth), and neonatal outcomes, including birth weight, small for gestational age (SGA), Apgar score and gestational age at delivery.

### Study population

We used a prospective cohort of 2252 pregnant women in the private mother-child health Budi Kemuliaan Hospital and its branch (Budi Kemuliaan Petojo) in Jakarta, Indonesia. The hospital provides secondary care on maternal health, while its branch focuses on primary care services. Women who were recruited were, therefore, representative of the pregnant women population in an urban setting of a developing country. Pregnant women were recruited during their first regular visit for antenatal care (ANC) between July 2012 and October 2014. All women who attended clinic visits were invited and asked to sign written informed consent. Participants were examined and interviewed by midwives according to standard clinical care and followed up until delivery.

After enrolment, information regarding personal affairs, medical status and clinical information was obtained through interviews by midwives at ANC visits. This included socio-economic background of women and partners, women’s medical history (including previous surgery, medication), current pregnancy (last menstrual period (LMP), pre-pregnancy weight), obstetrical history (parity, previous morbidity during pregnancy, previous mode(s) of delivery), and family history of disease. Clinical information at each ANC visit included weight of the mother, blood pressure, temperature, occurrence of hyperemesis gravidarum, and presence of proteinuria.

### Hyperemesis gravidarum exposure measurement

Hyperemesis gravidarum was diagnosed by midwives during routine ANC visits. Details about duration of complaints, weight loss, metabolic disturbances and associated hospitalization were recorded. For analysis, women were classified as those without, with mild or with severe hyperemesis gravidarum (women with >5% weight loss compared to pre-pregnancy weight). Only women with hyperemesis diagnosed in the first or second trimester were included [34].

### Outcome measurements

Hypertensive disorders of pregnancy were classified by International Society for the study of Hypertension in Pregnancy (ISSHP) definitions [35]. Gestational hypertension was defined as systolic blood pressure of 140 mmHg or more and/or diastolic blood pressure of 90 mmHg or more at two occasions in a woman with no hypertension prior to 20 weeks of gestation. In women with gestational hypertension, proteinuria was defined as 2+ dipstick in random urine samples. Preeclampsia was diagnosed if women with gestational hypertension had proteinuria [36] or if there were also one or more convulsions (eclampsia) [37]. Due to the low incidence of eclampsia, we analyzed eclampsia patients combined with the preeclampsia group. Chronic hypertension was defined as blood pressure exceeding 140/90 mmHg before pregnancy or before 20 weeks gestation, [38] and only when found elevated at ≥ 2 occasions. Women who were diagnosed with gestational hypertension or preeclampsia received treatment according to the standard hospital protocols.

Miscarriage was defined as fetal loss before 23 weeks of pregnancy and/or weighing up to 500 g, and stillbirth as birth of a baby with no signs of life at or after 28 weeks of gestation [39]. Small for Gestational Age (SGA) was defined as birth weight at a particular gestational age was below the 10th percentile of United States National Reference for Fetal Growth [40]. Low Birth Weight (LBW) was a birth weight below 2500 g [41]. Apgar scores were measured at 1, 5, and 10 min after birth [42]. Reported outcome for Apgar score was the score 5 min postpartum. Prematurity was defined as birth before 37 weeks of gestation. Gestational age at delivery was calculated in days by subtracting the first day of last menstrual period (LMP) from the date of admission to delivery room/operation theatre. Confirmation of gestational age using ultrasound scan was not done since only limited women had access to this examination. Nine women with gestational duration > 46 weeks (probably due to inaccuracy of gestational age calculation), were excluded.

After birth, birth weight and placental dimensions (weight, length, and width) were measured using a standardized method by midwives. The placenta-to-birth weight ratio was calculated [28, 31]. After discharge from hospital, active follow-up was terminated. Analyses on neonatal outcomes were based on singleton live birth pregnancies, and therefore multiple pregnancies, stillbirths and miscarriages were excluded from analysis.

### Confounding variables

Analyses of the associations between hyperemesis and placental dysfunction disorders were adjusted for socio-economic status (family income), second-hand smoking exposure, maternal age at delivery, gravidity, and pre-pregnancy body mass index (BMI).

### Data analysis

Baseline analysis was stratified by hyperemesis diagnosis (severe/mild/no hyperemesis), and differences between groups evaluated with Chi-square, one-way-ANOVA or Kruskal-Wallis tests where appropriate. For skewed data, we reported median and interquartile range (IQR). Main results were expressed as crude and adjusted linear regression coefficients and odds ratios from logistic regression, with corresponding 95% confidence intervals and p-values in tables. All statistical analyses were run using IBM SPSS (version 22 for Mac).

## Results

### Study population

**Fig. 1:**
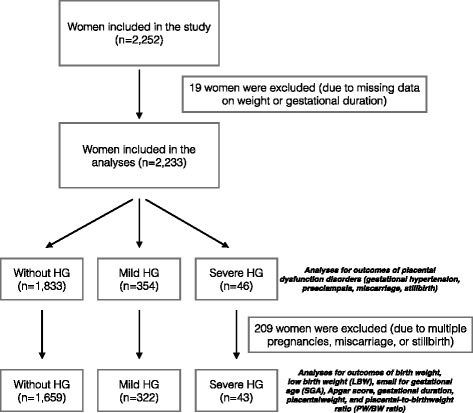
Flowchart of subject selection

Of 2252 participants, 400 were diagnosed with hyperemesis gravidarum (18.9%). Of the diagnosed, 94 experienced weight loss varying from 1 to 13 kg. There were 1833 women without, 354 with mild and 46 women with severe hyperemesis (weight loss > 5%). Subject selection is shown in Fig. 1. The mean age of participants was 28.3 years, 27% were primigravida.

Table 1 shows the baseline characteristics. The estimated total family income was used as a proxy of socio-economic status of the women and was categorized into 4 categories according to its distribution. We found that family income categories differed statistically significantly across women from different exposure groups. Women with hyperemesis also attended the first ANC visit earlier in gestation than women without hyperemesis.

**Table 1:** Baseline characteristics of pregnant women participating in our cohort by HG exposure status

	HG exposure			*P*
	No HG (*n* = 1833)	Mild HG (*n* = 354)	Severe HG (*n* = 46)	
Demographic characteristics				
Age (years) Median (IQR)	28.09 (8.7)	28.13 (7.4)	28.36 (9.1)	0.62
Women’s education^a^				0.15
*Low*	413 (22.6)	73 (20.6)	10 (22.2)	
*Intermediate*	1123 (61.4)	208 (58.8)	31 (68.9)	
*High*	292 (16.0)	73 (20.6)	4 (8.9)	
Partner’s education^a^				0.23
*Low*	323 (17.7)	46 (13.0)	9 (20.0)	
*Intermediate*	1199 (65.6)	241 (68.1)	30 (66.7)	
*High*	305 (16.7)	67 (18.9)	6 (13.3)	
Family income^b^				0.01*
*< 72 USD*	197 (10.8)	26 (7.3)	5 (10.9)	
*72–180 USD*	799 (43.6)	169 (47.7)	18 (39.1)	
*180–360 USD*	529 (28.9)	126 (35.6)	16 (34.8)	
*> 360 USD*	124 (6.8)	17 (4.8)	3 (6.5)	
*Refused to answer*	182 (9.9)	16 (4.5)	4 (8.7)	
Health characteristics				
Pre-pregnancy BMI Median (IQR)	22.0 (5.5)	22.2 (5.5)	23.2 (6.6)	0.59
Chronic hypertension	27 (1.5)	5 (1.4)	0	0.71
Type 2 diabetes	11 (0.6)	2 (0.6)	0	0.87
Partner’s smoking status				0.12
*Smokes daily*	394 (48.6)	70 (50.7)	8 (36.4)	
*Smokes occasionally*	113 (13.9)	12 (8.7)	1 (4.5)	
*Doesn’t smoke*	304 (37.5)	56 (40.6)	13 (59.1)	
Obstetrics characteristics				
Primigravida	480 (38.0)	112 (41.5)	12 (34.3)	0.50
Gestational age (weeks) at first visit Median (IQR)	16 (20)	9 (4.50)	10 (5.0)	0.00*
Reported complications in previous pregnancy				
*No*	1070 (84.6)	233 (86.0)	29 (82.9)	0.80
*IUGR/SGA*	3 (0.2)	0	0	0.70
*Hypertensive disorder in pregnancy*	44 (2.4)	11 (3.1)	1 (2.2)	0.74
*Miscarriage*	136 (7.4)	28 (7.9)	2 (4.3)	0.69
Female baby	651 (46)	133 (46.5)	22 (59.5)	0.27
Multiple pregnancy	27 (1.9)	1 (0.4)	0	0.12
Mode of delivery				0.08
*Vaginal*	871 (61.3)	168 (58.9)	24 (64.9)	
*Instrumental*	33 (3.1)	11 (3.9)	4 (10.8)	
*Caesarean section*	506 (35.6)	106 (37.2)	9 (24.3)	

*Abbreviations*: *HG* Hyperemesis gravidarum, *USD* United States Dollar, *IUGR* intra uterine growth restriction, *SGA* small for gestational age, *IQR* inter-quartile range. Results are median (inter-quartile range) or numbers (percentage); For continuous outcome variables Kruskall-Wallis test was used, for categorical variables, Pearson’s chi-square test

^a^ *Low education* = no education, elementary school, junior high school; *Intermediate education* = senior high school or above; *High education* = university

^b^Mean monthly total family income, estimated by the patient

*P <0,05

### Hyperemesis gravidarum (HG) exposure and outcomes

Tables 2, 3 and 4 show associations between hyperemesis and placental dysfunction disorders, neonatal outcomes and placental measures. Mild and severe hyperemesis were not associated with placental dysfunction disorders. After adjustment, babies of women with severe hyperemesis were on average 172 g lighter at birth than unexposed babies. No associations were found for SGA and LBW offspring, or other adverse neonatal outcomes. Women with mild hyperemesis had slightly lighter placentas while women with severe hyperemesis had heavier placentas, although none were statistically significant. Likewise, placental-weight-to-birth weight ratios (PW/BW ratio) were lower with mild hyperemesis and higher with severe hyperemesis, although not statistically significant.

**Table 2:** Hyperemesis gravidarum severity groups and placental dysfunction disorders

	*n* (%)	Crude model		Adjusted model^a^	
		OR (95% CI)	*p*	OR (95% CI)	*p*
Gestational Hypertension					
No HG	242 (17.7)	Reference		Reference	
Mild HG	39 (14.5)	0.79 (0.55; 1.13)	0.20	0.87 (0.58; 1.31)	0.51
Severe HG	4 (10.8)	0.56 (0.20; 1.60)	0.28	0.64 (0.22; 1.87)	0.41
Preeclampsia					
No HG	77 (6.0)	Reference		Reference	
Mild HG	15 (5.8)	0.97 (0.55; 1.72)	0.92	0.91 (0.47; 1.76)	0.78
Severe HG	2 (5.4)	0.90 (0.21; 3.81)	0.89	0.99 (0.23; 4.34)	0.99
Miscarriage					
No HG	136 (7.4)	Reference		Reference	
Mild HG	28 (7.9)	1.07 (0.70; 1.64)	0.75	1.17 (0.60; 2.70)	0.64
Severe HG	2 (4.3)	0.57 (0.13; 2.37)	0.44	-	-
Stillbirth					
No HG	12 (0.8)	Reference		Reference	
Mild HG	3 (1.0)	1.24 (0.35; 4.43)	0.74	1.41 (0.37; 5.40)	0.62
Severe HG	1 (2.7)	3.26 (0.41; 25.72)	0.26	4.18 (0.50; 35.10)	0.19

*Abbreviations*: *HG* hyperemesis gravidarum, *OR* odds ratio

^a^Adjusted model: adjusted for socio-economic status (as reflected by income), smoking status, gravidity, maternal age and pre-pregnancy BMI

**Table 3:** Hyperemesis gravidarum severity groups and neonatal outcome

	Values	Crude model		Adjusted model^a^	
		Coefficients (95% CI)^b^	*p*	Coefficients (95% CI)^b^	*p*
Birthweight (g)^c,d^					
No HG	3100 (476.6)	Reference		Reference	
Mild HG	3116 (464.4)	16.36 (-44.82; 77.54)	0.60	-4.17 (-68.36; 60.02)	0.90
Severe HG	3046 (485.7)	-53.86 (-211.15; 103.43)	0.50	-171.72 (-333.26; -10.18)	0.04
SGA^c^					
No HG	212 (16.6)	Reference		Reference	
Mild HG	54 (19.8)	1.24 (0.89; 1.73)	0.20	1.29 (0.87; 1.91)	0.20
Severe HG	6 (17.6)	1.08 (0.44; 2.64)	0.87	1.74 (0.68; 4.44)	0.25
LBW^c^					
No HG	101 (7.4)	Reference		Reference	
Mild HG	21 (7.5)	1.03 (0.63; 1.67)	0.92	1.36 (0.74; 2.51)	0.32
Severe HG	2 (5.6)	0.74 (0.18; 3.13)	0.68	1.44 (0.33; 6.33)	0.63
Gestational age at delivery (in days)^e^					
No HG	274 (17)	Reference		Reference	
Mild HG	275 (13)	0.27 (-2.22; 2.75)	0.83	-0.03 (-2.60; 2.54)	0.98
Severe HG	276 (19)	0.48 (-5.99; 6.96)	0.88	-2.20 (-8.81; 4.42)	0.52
Apgar score^c,e^					
No HG	9 (0)	Reference		Reference	
Mild HG	9 (0)	-0.06 (-0.17; 0.04)	0.24	-0.08 (-0.19; 0.03)	0.15
Severe HG	9 (0)	-0.06 (-0.35; 0.23)	0.67	-0.09 (-0.39; 0.21)	0.56

*Abbreviations*: *CI* confidence interval, *HG* hyperemesis gravidarum, *g* grams, *LBW* low birthweight, *SGA* small for gestational age

^a^Adjusted model: adjusted for socio-economic status (as reflected by income), smoking status, gravidity, maternal age and pre-pregnancy BMI

^b^Results are expressed as linear regression coefficients (95% CI) in continuous outcomes or OR (95% CI) from logistic regression in case of dichotomous outcomes

^c^Multiple pregnancies, stillbirths and miscarriages were excluded from the analyses

^d^Values are reported as mean and standard deviation (SD)

^e^Values are reported as median and inter-quartile range (IQR)

**Table 4:** Hyperemesis gravidarum severity groups and placental measures

	Values (SD)	Crude model		Adjusted model^a^	
		Coefficients (95% CI)^b^	*p*	Coefficients (95% CI)^b^	*p*
Placental weight (g)^c^					
No HG	536 (145.2)	Reference		Reference	
Mild HG	530 (102.3)	-5.98 (-42.92; 30.95)	0.75	-6.05 (-41.25; 29.15)	0.74
Severe HG	547 (98.4)	10.73 (-126.91; 148.38)	0.88	47.76 (-97.62; 193.14)	0.52
PW/BW ratio^c^					
No HG	0.18 (0.05)	Reference		Reference	
Mild HG	0.18 (0.05)	0.001 (-0.01; 0.01)	0.89	0.002 (-0.01; 0.02)	0.72
Severe HG	0.19 (0.05)	0.02 (-0.03; 0.06)	0.54	0.04 (-0.01; 0.09)	0.15

*Abbreviations*: *CI* confidence interval, *HG* hyperemesis gravidarum, *PW/BW ratio* placenta-to-birthweight ratio, *g* grams

^a^Adjusted model: adjusted for socio-economic status (as reflected by income), smoking status, gravidity, maternal age and pre-pregnancy BMI

^b^Results are expressed as linear regression coefficients (95% CI)

^c^Multiple pregnancies, stillbirths and miscarriages were excluded from the analyses

Additional analyses regarding the effect of absolute and relative weight change during the early part of pregnancy were also conducted within women with hyperemesis gravidarum. However, no associations were found between early pregnancy weight change with later placental dysfunction disorders and neonatal or placental outcomes.

## Discussion

This study shows that severe hyperemesis gravidarum is associated with a significant decrease in birth weight, but no associations between HG and gestational hypertension, preeclampsia or other placental dysfunction disorders were observed. No links were found between hyperemesis gravidarum and neonatal outcomes, such as SGA and LBW.

Strengths of this study comprise the prospective design and the number of included women. This allowed for assessing both first and second trimester effects of hyperemesis gravidarum on neonatal outcomes and particularly birth weight, to give an impression of effects on the placenta, and evaluate early pregnancy weight change as a possible mediator of the effects. However, given the low incidence of placental dysfunction disorders, our study may have been too small for definite inference. Of participants, 18.9% had some form of hyperemesis, which is very high, but our estimates of severe hyperemesis do fit previous reports. Notably, women were recruited from a referral institute for mother and child health, and indeed some with hyperemesis were referred earlier in pregnancy. Although the data about indication for referral was not available, we believe that specific referrals for hyperemesis, particularly those in early pregnancy, were for that complaint only, and not for some associated expectation of higher risk for adverse maternal or neonatal outcome, such that selection bias is unlikely. This was supported by our finding that hyperemetic pregnant women have comparable risks for obstetric complications as compared to women without hyperemesis, which include pre-pregnancy BMI, chronic hypertension and type 2 diabetes, and previous obstetric complications. Missing information increased with longer follow-up, but was largely due to women who were temporarily provided antenatal care services and then referred back to primary care, a routine that we consider unrelated to the association of interest. Our self-report questionnaire information may contain measurement error, but as patients were unaware of the study aim, such error was likely random.

Our results do not show that severe hyperemesis gravidarum increases the risk of placental dysfunction disorders. A link between hyperemesis gravidarum and placental dysfunction disorders was first suggested in a 1991 case-control study reporting a 1.6 times higher risk for preeclampsia in women with severe vomiting [43]. Consistent with our findings, previous studies also showed no increased risks of placenta dysfunction disorders with exposure to hyperemesis [44, 45]. The largest cohort to date with 1,155,033 pregnancies, of which 13,287 were complicated by hyperemesis leading to hospital admission, showed a slightly increased risk for preeclampsia, and a higher risk for pre-term preeclampsia when hyperemesis occurred in the second trimester [15]. It has been suggested that differences in outcome in hyperemetic pregnancies are explained by maternal characteristics such as (gestational) hypertension, (gestational) diabetes, and primiparity [46]. A general problem in comparing findings on relations between hyperemesis and placental dysfunction disorders is the lack of a widely accepted definition of ‘severe’ hyperemesis. Working criteria range from hyperemesis requiring hospitalization or vomiting with associated metabolic disturbances to classifications purely based on caregiver’s diagnosis. In this study, the presence of (maternal) weight loss of more than 5% (compared to the weight prior to diagnosis) was used as criterion to classify severity of hyperemesis. The measured relative weight loss provided a more objective cut off for hyperemesis severity which is also generalizable to all pregnant women of different weight.

We showed a (adjusted) 172 g lower birth weight in offspring of women with severe hyperemesis, which was not explained by gestational duration. This was in accordance with several previous reports [22, 46–49]. In this study, we did not find any association between hyperemesis and SGA or LBW, although these appeared more common in women with severe hyperemesis. Although exposed babies were born smaller, it appeared that their chances of passing the threshold of the 10th percentile for the gestational age (SGA) or 2500 g for birth weight (LBW) were equal to the non-exposed. This agrees with several previous reports, including the largest study on neonatal outcome to date [21, 27, 50]. Differences in exposure definition and classification of its severity may contribute to the variation of findings.

Previous research on the association between hyperemesis and placental measures is limited. In the present study, we detected no effects on placental weight and placenta-weight-to-birth weight ratio (PW/BW ratio). Women with severe hyperemesis, however, had heavier placentas and higher of PW/BW ratio, although not statistically significant. Heavier placentas and higher PW/BW ratio were previously reported, but only for female offspring [28]. Studies on famine and placental weight and PW/BW ratio suggest that with low caloric intake, the placenta compensatorily grows, probably to maintain adequate fetal nutrition [31, 32]. This might also occur with hyperemesis and, therefore, further research on the placenta is warranted; especially since there is evidence that compensatory growth of the placenta is associated with cardiovascular problems in later life [28, 51].

Further research requires clear HG definition and severity criteria. Large cohort studies will be needed to estimate relations between hyperemesis and placental dimensions, and rare outcomes, such as eclampsia and stillbirth. Conducting follow up studies of women with severe hyperemesis gravidarum will give a better insight in possible long term effects of hyperemesis on the health of children born out of hyperemetic pregnancies.

## Conclusions

Hyperemesis gravidarum is an invalidating disease in early pregnancy, associated with hospitalizations, use of medication and a poorer quality of life. However, our findings do indicate no relevant impact of hyperemesis gravidarum on placental dysfunction disorders.

## Abbreviations

ANC: Antenatal care
ANOVA: Analysis of variance
BMI: Body mass index
hCG: Human chorionic gonadotropin
HG: Hyperemesis gravidarum
ICD-9: The International Statistical Classification of Disease and Related Health Problems
IQR: Interquartile range
ISSHP: International Society for the study of Hypertension in Pregnancy
IUGR: Intra-uterine growth restriction
LBW: Low birth weight
LMP: Last menstrual period
PE: Preeclampsia
PW/BW ratio: Placental-weight-to-birth-weight ratio
SGA: Small for gestational age
